# Comparison of single-incision laparoscopic percutaneous extraperitoneal closure (SILPEC) and open repair for pediatric inguinal hernia: a single-center retrospective cohort study of 2028 cases

**DOI:** 10.1007/s00464-017-5472-6

**Published:** 2017-06-08

**Authors:** Hizuru Amano, Yujiro Tanaka, Hiroshi Kawashima, Kyoichi Deie, Michimasa Fujiogi, Keisuke Suzuki, Kaori Morita, Tadashi Iwanaka, Hiroo Uchida

**Affiliations:** 1 Department of Pediatric Surgery, Saitama Children’s Medical Center, 1-2, Shintoshin, Chuo-ku, Saitama-city, Saitama, 330-8777 Japan; 2 Department of Pediatric Surgery, Graduate School of Medicine, The University of Tokyo, 7-3-1, Hongo, Bunkyo-ku, Tokyo, 113-8655 Japan; 3 Department of Pediatric Surgery, Nagoya University Graduate School of Medicine, 65 Tsurumai, Showa, Nagoya, 466-8550 Japan

**Keywords:** Pediatric, Inguinal hernia, Laparoscopic inguinal hernia repair, Laparoscopic percutaneous extraperitoneal closure, Reduced port surgery, Contralateral metachronous inguinal hernia

## Abstract

**Backgroud:**

Recently, laparoscopic percutaneous extraperitoneal closure (LPEC) has gained increased popularity for pediatric inguinal hernia repair. To improve cosmesis, we developed single incision LPEC (SILPEC). The aim of this study was to assess the safety and feasibility of SILPEC compared with traditional open repair (OR).

**Methods:**

This was a single-center retrospective cohort study of 2028 children who underwent inguinal hernia repair between April 2005 and August 2014. Nine hundred and ninety-five patients underwent OR and 1033 patients underwent SILPEC. Medical records were reviewed with respect to operative time, recurrence, incidence of contralateral metachronous inguinal hernia (CMIH), and complications. Patient satisfaction with cosmetic result was also investigated using questionnaires sent by mail.

**Results:**

All SILPEC procedures were completed without conversion. Operative time was longer in the SILPEC group than in the OR group for both unilateral and bilateral surgery regardless of sex (unilateral male: *p* = 0.0006, unilateral female: *p* < 0.0001, bilateral male: *p* < 0.0001, bilateral female: *p* < 0.0001). There was no statistically significant difference in recurrence rate (*p* = 0.43). The incidence of CMIH was significantly higher in the OR than in the SILPEC group (*p* < 0.0001). No postoperative testicular atrophy was found in either group. There was no statistically significant difference in ascending testis (*p* = 0.09), but the frequency of surgical site infection was higher in the SILPEC than in the OR group (*p* = 0.0013). According to the questionnaire, operative scar was more invisible in the SILPEC than in the OR group (*p* < 0.0001), but both procedures had equally high levels of satisfaction for cosmetic results (*p* = 0.58).

**Conclusion:**

SILPEC proved to be a safe and feasible procedure compared with OR with an equally low recurrence rate, more effectiveness for preventing CMIH, and more invisible scar.

In pediatric surgery, inguinal herniorrhaphy is one of the most common surgical procedures. Recently, laparoscopic procedures for pediatric inguinal hernia repair have gained increased popularity, and numerous techniques have been reported [1–4]. Laparoscopic percutaneous extraperitoneal closure (LPEC), first described by Takehara et al. [5], has been now widely accepted as one of the most simple and reliable methods for pediatric inguinal hernia repair. We introduced LPEC at our institution in February 2007 and developed single-incision LPEC (SILPEC) in December 2009 as a less invasive and more cosmetically appealing technique [6]. Although there are some comparative reports of conventional open repair (OR) and LPEC [7–9], none has compared OR and SILPEC. We performed a retrospective cohort study including 2028 children who underwent inguinal hernia repair to compare the safety and efficacy of OR and SILPEC at Saitama Children’s Medical Center in Japan.

## Materials and methods

### Study design

This was a single-center, retrospective cohort study including 2028 children who underwent inguinal hernia repair between April 2005 and August 2014 at Saitama Children’s Medical Center in Japan. To assess safety and efficacy of SILPEC, it was compared with traditional OR. We started LPEC in February 2007, and developed SILPEC in December 2009. After SILPEC was introduced, patients underwent either OR or SILPEC based on their preferences after providing informed consent. Medical records were reviewed with respect to operative time, recurrence, incidence of contralateral metachronous inguinal hernia (CMIH), and complications. Patient satisfaction with the cosmetic result was also investigated through questionnaires sent to parents by mail on February 2016. Visibility of scar, umbilical protrusion deformity after SILPEC, and cosmetic satisfaction were estimated according to a 5-point scale (Visibility of scar: 1 = well visible−5 = not visible at all; Umbilical deformity after SILPEC: 1 = worse−5 = better; Cosmetic satisfaction: 1 = not satisfied at all –5 = very satisfied).

All cases who underwent both OR and SILPEC were clinically diagnosed as an indirect inguinal hernia. Criteria for enrollment included indirect inguinal hernia, associated hydrocele, or incarcerated hernia. Exclusion criteria included patients with ascending testis, and those who underwent herniorrhaphy of either same or contralateral side. Patients who underwent other procedures such as umbilicoplasty at the same time with herniorrhaphy were excluded from the analysis of the operative time.

Diagnosis was made by examination by a surgeon or ultrasonography in the outpatient clinic. Patients were followed-up regularly in our outpatient clinic at 1 week and 6 months postoperatively to assess wound healing, in addition to the size and position of the testes in males. We informed parents to visit our institution again if they have any complaints after the end of the regular follow-up period. In this study, we sent the questionnaire to the parents and confirmed the clinical outcome, such as past surgical history of recurrence or CMIH at other institutions after the first surgery at our institution. The end of the follow-up period was February 2016 when the questionnaires were returned to us. In the case of unreturned questionnaires, the end of the follow-up period was considered to be the last consultation day at our outpatient clinic.

This study protocol was approved by the Ethical Committee at our institution and met the guidelines of the responsible governmental agency. Guardians of all participants gave written informed consent.

### Statistical analyses

Continuous variables were presented as mean ± standard deviation. Statistical analyses were performed using the *χ*2 test for categorical variables and the Mann–Whitney *U*-test for continuous variables. A *p*-value of less than 0.05 was considered to be statistically significant. Asterisks indicate statistical significance at different levels: ***if *p*-value < 0.001, **if *p*-value < 0.01, * if *p*-value < 0.05.

### Surgical procedures

### Open repair

OR was performed according to the technique described by Potts et al. [10], which was simple high ligation and possible removal of the hernial sac without elevating the structures of the cord, and without any plastic repair of the muscles or fascia of the inguinal region. Indirect sliding inguinal hernias of the ovary or tube were repaired based on the procedures described by Woolley [11].

### SILPEC

**Fig. 1:**
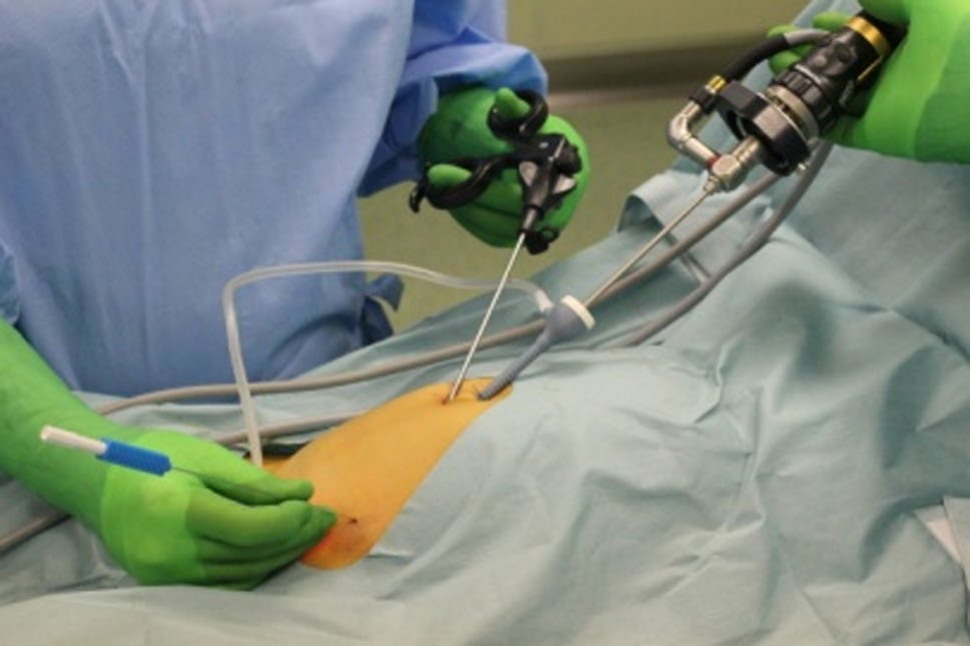
A camera port and a grasping forceps are inserted through the same umbilical incision

This procedure was described in our previous report [6]. Under general anesthesia, patients were placed in the supine position. A rectus sheath block was performed to relieve the postoperative umbilical pain. The viewing monitor was placed at the patient’s feet. The operator stood on the opposite side to the inguinal hernia, and the camera assistant stood on the other side. Through a 1.0 cm vertical umbilical incision, a 4 mm port for a 3 mm, 30° laparoscope was placed using an open technique. A 3 mm curved grasping forceps was inserted through the same umbilical incision with a different entrance (Fig. 1). A 19-gauge LPEC needle (Lapaherclosure™; Hakko Medical Co., Nagano, Japan), which has a wire loop to hold suture material at the tip, with non-absorbable suture material (2−0 polyester fibers; Wayolax, Matsuda Iuka Kogyo Company, Tokyo, Japan), was inserted at the midpoint of the affected side of the inguinal line. Using the LPEC needle and with the aid of the grasping forceps, the hernial sac was closed extraperitoneally with circuit suturing around the internal inguinal ring without any peritoneal gap, taking care to avoid injury to the vas deferens and spermatic vessels in males. The circuit suturing was tied extracorporeally, and the hernial sac was completely closed. Double ligation was introduced for cases over 5 years old and cases with hydrocele since July 2012. An asymptomatic contralateral internal inguinal ring was routinely observed, and prophylactic surgery was performed in patients with a contralateral patent processus vaginalis (cPPV). Closure of the peritoneum and fascia was performed for the umbilical incision. In patients with hydrocele, the hydrocele was punctured from the scrotum.

## Results

Between April 2005 and August 2014, 995 patients underwent OR and 1033 patients underwent SILPEC at Saitama Children’s Medical Center in Japan. Patients’ characteristics are listed in Table 1. Sex ratio (males: females) was 632:363 in the OR group and 488:545 in the SILPEC group (*p* < 0.0001***). Mean age at operation was 48.8 ± 36.0 months in the OR group and 49.0 ± 36.2 months in the SILPEC group (*p* = 0.98). Mean body weight at operation was 15.8 ± 8.1 kg in the OR group and 17.0 ± 8.8 kg in the SILPEC group (*p* = 0.0002***). The mean follow-up period was 49.3 ± 50.5 months in the OR group and 29.1 ± 24.3 months in the SILPEC group (*p* < 0.048*).

**Table 1:** Patients’ characteristics (*n* = 2028)

Characteristics	OR (*n* = 995)	SILPEC (*n* = 1033)	*p*-value
Sex (male:female)	632:363	488:545	<0.0001***^,¶^
Age (months)	48.8 ± 36.0	49.0 ± 36.2	0.98^,†^
Body weight (kg)	15.8 ± 8.1	17.0 ± 8.8	0.0002***^,†^
Preoperative laterality (right/left/bilateral)	540/361/94	408/551/74	<0.0001***^,¶^
Follow-up period (months)	49.3 ± 50.5	29.1 ± 24.3	0.048*^,†^
Associated morbidities			
Umbilical hernia	9/995	44/1033	<0.0001***^,¶^
Hydrocel(/preoperative symptomatic sides)	79/1089	120/1107	0.0034**^,¶^
Sliding hernia with ovary and ovarian duc(/preoperative symptomatic sides in females)	51/410	50/536	0.12^¶^
Intestinal incarceratio(/preoperative symptomatic sides)	86/1089	68/1107	0.11^¶^

Data are presented as mean ± standard deviation or numbers as indicated

^¶^Chi-squared test, ^†^Mann–Whitney *U*-test

*Asterisks* indicate statistical significance at different levels: ***if *p*-value < 0.001, **if *p*-value < 0.01, *if *p*-value < 0.05

In the OR group, 901 of 995 patients clinically diagnosed with unilateral inguinal hernia (540 right-sided, and 361 left-sided inguinal hernia) underwent unilateral surgery, and 94 of 995 patients clinically diagnosed with bilateral inguinal hernia underwent bilateral surgery. Thus, a total of 1089 internal inguinal rings (901 unilateral, and 94 bilateral) were closed in the 995 patients in the OR group. On the other hand, in the SILPEC group, 959 of 1033 patients were clinically diagnosed as having unilateral inguinal hernia (408 right-sided and 551 left-sided inguinal hernias), and 40% (380/959) of them were confirmed to have a cPPV intraoperatively and underwent simultaneous prophylactic surgery. Thus, in the SILPEC group, 579 of 1033 patients underwent unilateral surgery and 454 of 1033 underwent bilateral surgery. A total of 1487 internal inguinal rings (579 unilateral and 454 bilateral including 380 cPPVs) were closed in the 1033 patients of the SILPEC group.

Umbilical hernia was simultaneously treated in 9 (0.9%) patients of OR group and in 44 (4.3%) patients of SILPEC group (*p* < 0.0001***). Hydrocele was associated with preoperative symptomatic sides in 7.3% of patients in the OR group and 10.8% of those in the SILPEC group (*p* = 0.0034**). Sliding hernia of ovary and duct was associated with preoperative symptomatic sides in 12.4% of female patients in the OR group and 9.3% of those in the SILPEC group (*p* = 0.1247). Intestinal incarceration was associated with the preoperative symptomatic sides in 7.9% of patients in the OR group and 6.1% of those in the SILPEC group (*p* = 0.1075).

Operative time was compared except for cases with an additional operation including umbilicoplasty (Table 2). Operative time was significantly prolonged in the SILPEC group for both unilateral and bilateral surgery regardless of sex. In male unilateral cases, the mean operative time was significantly shorter in the OR group (23.3 ± 12.0 min) than in the SILPEC group (32.8 ± 9.2 min) (*p* = 0.0006***). In female unilateral cases, mean operative time was significantly shorter in the OR group (17.2 ± 9.6 min) than in the SILPEC group (30.4 ± 9.1 min) (*p* < 0.0001***). In male bilateral cases, mean operative time was significantly shorter in the OR group (42.0 ± 19.9 min) than in the SILPEC group (45.5 ± 12.7 min) (*p* < 0.0001***). In female bilateral cases, the mean operative time was significantly shorter in the OR group (31.4 ± 9.2 min) than in the SILPEC group (42.2 ± 11.6 min) (*p* < 0.0001***).

**Table 2:** Comparison of the operative time in the OR and SILPEC groups

	OR (min)	SILPEC (min)	*P*-value
Unilateral			
Male	23.3 ± 12.0	32.8 ± 9.2	^†^0.0006***
Female	17.2 ± 9.6	30.4 ± 9.1	^†^<0.0001***
Bilateral			
Male	42.0 ± 19.9	45.5 ± 12.7	^†^<0.0001***
Female	31.4 ± 9.2	42.2 ± 11.6	^†^<0.0001***

Data are presented as mean ± standard deviation

^†^Mann–Whitney U-test

All SILPEC procedures were completed laparoscopically without open conversion or requiring additional skin incision. Intra- and postoperative complications are listed in Table 3. No testicular atrophy was detected in either group. Inguinal swelling occurred statistically more frequent in the OR group (3.2%; 22/681 sides of surgery performed in males) than in the SILPEC group (0.1%; 1/716 sides of surgery performed in males) [*p* < 0.0001***]. There was no statistically significant difference in postoperative ascending testis between the OR (0.7%; 5/681 sides of surgery performed in males) and the SILPEC groups (0.1%; 1/716 sides of surgery performed in males) [*p* = 0.09]. Incidence of surgical site infection requiring oral antibiotics (Grade II) was significantly lower in the OR group (0.2%; 2/995 cases) than in the SILPEC group (1.5%; 16/1033 cases) [*p* = 0.0013**]. Intraoperative bleeding occurred in 4 cases in the SILPEC group, but hemostasis was established with manual extracorporeal compression in all cases.

**Table 3:** Comparison of the complications in the OR and SILPEC groups

	OR	SILPEC	*p-*value
Recurrence (/preoperative symptomatic sides)	2/1089 (0.2%)	4/1107 (0.4%)	0.43^¶^
CMIH (/preoperative asymptomatic sides)	44/901 (4.9%)	3/959 (0.3%)	<0.0001***^,¶^
Bleeding (/sides of surgery performed)	0/1089	4/1497 (0.3%)	0.09^¶^
Inguinal swelling (/sides of surgery performed in males)	22/681 (3.2%)	1/716 (0.1%)	<0.0001***^,¶^
Ascending testis (/sides of surgery performed in males)	5/681 (0.7%)	1/716 (0.1%)	0.09^¶^
Testicular atrophy (/sides of surgery performed in males)	0/681	0/716	–
Surgical site infection requiring oral antibiotics	2/995 (0.2%)	16/1033 (1.5%)	0.0013**^,¶^

Data are presented as numbers (percentage)

*CMIH* contralateral metachronous inguinal hernia

^¶^Chi-squared test

**Fig. 2:**
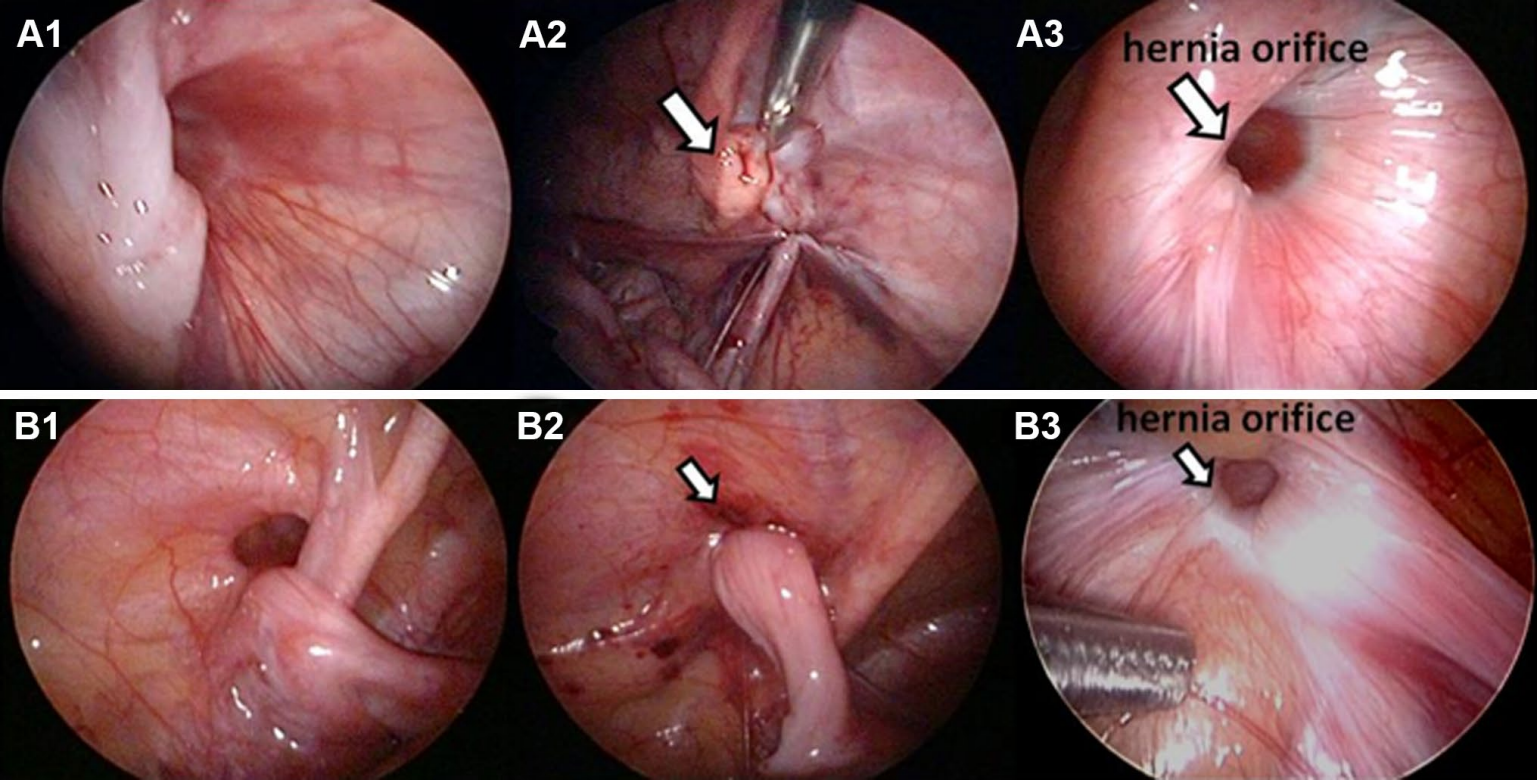
Findings at reoperation after SILPEC with single ligation.** A** Male patient aged 1 year and 5 months. *A1* Operative findings at the first surgery. *A2* Adjacent tissues (*white arrow*) were involved and ligated together after high ligation at the first surgery. *A3* Knot was loosened at the reoperation 5 months after the first surgery **B** Female patient aged 8 months. *B1*. Operative findings at the first surgery. *B2* The round ligament (*white arrow*) was thickened after high ligation at the first surgery. *B3* Knot was loosened at the reoperation 19 months after the first surgery

Recurrence rate was 0.2% (2/1089 preoperative symptomatic sides) in the OR group, and 0.4% (4/1107 preoperative symptomatic sides) in the SILPEC group (*p* = 0.43). Two recurrent cases in the OR group were a male and a female without sliding hernia or intestinal incarceration, respectively. Both recurrences occurred within a year. During each open reoperation, a knot was seen outside the hernial sac, so high ligation was performed again. The recurrent cases in the SILPEC group are listed in Table 4. Recurrences after SILPEC occurred within 2 years with hydrocele. The cause of recurrence in three patients with single ligation at the first surgery was loosening of the knot (Fig. 2). All 3 recurrent cases after SILPEC with single ligation underwent SILPEC with double ligation. Since the introduction of double ligation, no recurrence due to loosening of the knot was recognized. The cause of recurrence in one hydrocele patient with double ligation was non-communicating hydrocele. After complete closure of the hernial sac was confirmed laparoscopically at reoperation, open repair with removal of the hernial sac was performed in this case.

**Table 4:** Recurrent cases in the SILPEC group

Primary operation						Recurrence		Reoperation	
Age	Sex	Diagnosis	Sliding hernia	Incarceration	Ligation	Time	Symptom	Operative finding	Procedure
1 year 5 months	M	Inguinal hernia	–	–	Single	1.5 months	Hydrocele	Loosening of the knot	SILPEC (double ligation)
2 year 6 months	M	Hydrocele	–	–	Single	20 months	Hydrocele	Loosening of the knot	SILPEC (double ligation)
8 months	F	Inguinal hernia	–	–	Single	1 month	Hydrocele	Loosening of the knot	SILPEC (double ligation)
11 year 11 months	M	Hydrocele	–	–	Double	0.8 month	Hydrocele	Non-communicating hydrocele	Potts after laparoscopic exploration

The incidence of postoperative CMIH was 4.9% (44/901 preoperative asymptomatic sides) in the OR group and 0.3% (3/959 preoperative asymptomatic sides) in the SILPEC group (*p* < 0.0001***).

**Fig. 3:**
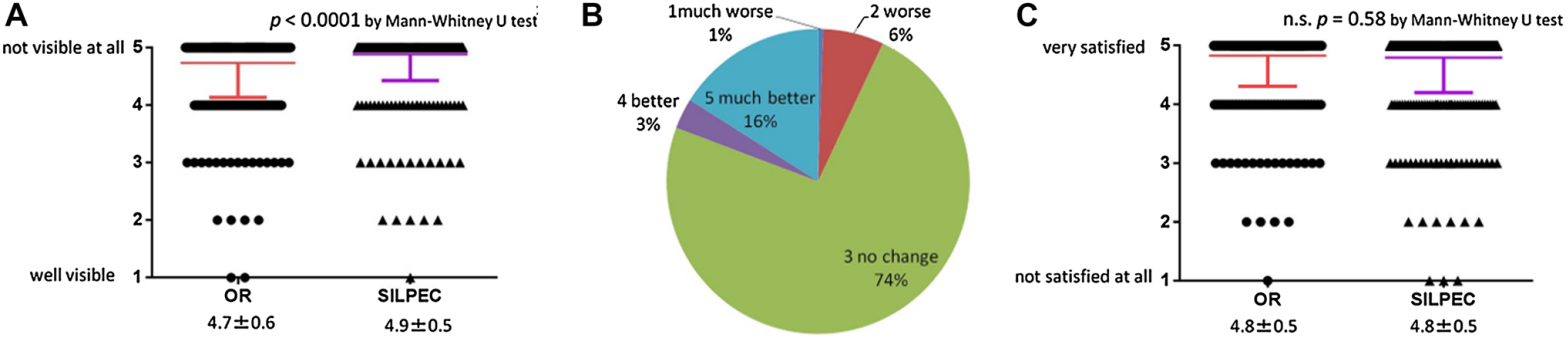
Patient satisfaction with cosmetic result through the questionnaires sent by mail. **A** Visibility of scar. **B** Deformity of umbilicus after SILPEC. **C** Cosmetic satisfaction

Questionnaires concerning patients’ satisfaction with cosmetic result were sent to 2028 parents and returned by 1095 responders (53%); 490 patients from the OR group (48.9%) and 605 patients from the SILPEC (57.6%). The mean rating for scar visibility on a 5-point scale (1 = well visible-5 = not visible at all) was 4.7 ± 0.6 in the OR group, and 4.9 ± 0.5 in the SILPEC group (*p* < 0.0001***) (Fig. 3A). For patients in the SILPEC group who did not undergo umbilicoplasty, 7% reported that the umbilicus changed to the worse, 19% reported that the umbilicus changed to the better (Fig. 3B), and the rest of them reported no change in the umbilicus. The mean rating for cosmetic satisfaction on a 5-point scale (1 = not satisfied at all-5 = very satisfied) was 4.8 ± 0.5 in the OR group, and 4.8 ± 0.5 in the SILPEC group (*p* = 0.58) (Fig. 3C).

## Discussion

Inguinal hernia is one of the most common abnormalities of the inguinoscrotal region due to failure of closure of the processus vaginalis in children. Open repair with high ligation has been a standard procedure [10]. Recently, laparoscopic approach has gained increased popularity due to its advantages that include excellent operative field, prophylactic surgery of the contralateral side and preventing injury to the vas deferens and spermatic vessels [1–4]. Although there are numerous techniques for laparoscopic procedures, LPEC, as described by Takehara et al. [5], is one of the most simple and reliable methods, because of minimal dissection, less complications, comparable recurrence rates, and improved cosmetic results [5, 7–9].

In the LPEC procedure, the hernial sac is closed extraperitoneally using a 19-gauge LPEC needle with the aid of a grasping forceps [5]. However, conventionally, LPEC necessitate two skin incisions for a camera and a grasping forceps. In order to reduce the incisions for better cosmesis, we devised a technical method to insert a grasping forceps through a different entrance in the same umbilical incision for a camera port. We named this new method SILPEC [6]. To avoid the interference between the camera and the grasping forceps during SILPEC, we developed a curved grasping forceps, which makes the procedure easier. We have previously reported its safety and feasibility compared with LPEC [6], but no reports have described large case–control studies comparing OR and SILPEC. We have already performed over 1000 cases of SILPEC, so we conducted a comparative study of 2028 children who underwent inguinal hernia repair by OR or SILPEC. We evaluated not only the safety and feasibility of SILPEC compared with OR, but also patients’ satisfaction with the cosmetic result through the questionnaires, which have hardly been statistically estimated in previous reports. Moreover, cause of recurrence in SILPEC was speculated according to the reoperative findings. In our study, the operative time was statistically longer in SILPEC than OR group for both unilateral and bilateral surgery regardless of sex. However, Miyake et al. reported that the operative time was significantly shorter in LPEC than OR group [7]. In our institution, during closure of the umbilical incision, meticulous care is taken not to leave umbilical deformity for better cosmesis. This maneuver might have resulted in a rather longer operative time. Considering the low dissatisfaction to umbilical deformity after SILPEC according to the questionnaires, this rather longer duration seems to be acceptable.

As for perioperative complications of these procedures, intraoperative bleeding, postoperative inguinal swelling, ascending testis, testicular atrophy, and surgical site infection have been reported. In our study, no postoperative testicular atrophy was recognized in both procedures. There was no significant difference in postoperative ascending testis, but inguinal swelling occurred more frequently in OR than SILPEC group. Inguinal swelling is thought to be caused by touching cord structures. Considering that injury to the reproductive system is the most frequent cause of infertility in children [12], SILPEC can be said to be less invasive to the vas deferens and spermatic vessels than OR, and a safer procedure. Previous reports also pointed out these advantages of laparoscopic procedures including the better visualization of vital cord structures, which makes dissection of these structures safer and easier, and limits dissection field to the peritoneal layer, with the vas deferens and cord untouched [1]. Furthermore, testicular complications, such as testicular atrophy and ascending testis, are also very crucial in males, so SILPEC is a safer procedure compared with OR in this perception as well. There was also no significant difference in intraoperative bleeding, with bleeding occurring in 4 SILPEC cases only. Hemostasis was achieved with manual extracorporeal compression in all cases. All four procedures were performed by less experienced surgeons. It appears that experienced surgeons have fewer patients developing intraoperative bleeding, and more practice can reduce bleeding in SILPEC. In this study, the frequency of surgical site infection requiring oral antibiotics (Grade II) was higher in SILPEC than OR group. In our institution, although the umbilicus was routinely cleaned meticulously before operation, it remained not clean due to the blot. Operative scar of SILPEC was more invisible than OR and the cosmetic satisfaction was equally high in both groups according to the questionnaire concerning patient satisfaction with cosmetic result. In this respect, the results regarding SSIs in the present study seem acceptable.

Recurrence rates were considerably low in both groups without significant difference. Recurrence occurred within 2 years in both groups. In SILPEC with single ligation, the causes of recurrence were loosening of the knots. According to the findings of reoperation, we speculated that the knots were loosened because of the thickened tissues (Fig. 2). In order to prevent recurrence, double ligation was introduced in July 2012 for the cases over 5 years old whose tissues seem to be thickened, and for patients with hydrocele who seem to be liable for recurrence and need watertight closure of the hernial sac. Since then, no recurrence due to loosening of the knot has been recognized yet. Double ligation seems to be effective for preventing recurrence (single ligation vs. double ligation: 3/859 preoperative symptomatic sides vs. 0/248 preoperative symptomatic sides). Previous articles reported that injury or skip of peritoneum might contribute to recurrence in LPEC [7–9]. In our institution, there were 2 recurrent cases (0.6%: 2/310 preoperative symptomatic sides in 293 patients who underwent LPEC) due to incomplete circuit suturing of the hernial sac with skipped or injured peritoneum soon after LPEC was introduced and before SILPEC was developed. However, no recurrence due to incomplete circuit suturing was seen in SILPEC. This suggests that recurrence due to incomplete circuit suturing can be prevented with more experience of laparoscopic procedures.

On the other hand, the incidence of CMIH was statistically higher in OR than in SILPEC group. In the SILPEC group, 40% of patients with clinical unilateral inguinal hernia had cPPV and underwent prophylactic surgery. Considering the low incidence of CMIH in the SILPEC group, prophylactic contralateral SILPEC seems to be effective for preventing CMIH. The effectiveness of laparoscopic repair for preventing CMIH was supported by some comparative studies of OR and laparoscopic repair [2–4, 6–9, 13]. Previous reports stated that cPPV was seen in 19.9–66% of cases [2, 5–9, 14]. The range of cPPV presence rates are wide because some cPPVs are hidden by a peritoneal slit or veil and hence difficult to be identified. In order to reduce the incidence of CMIH, careful observation for contralateral inguinal internal ring with the aid of a grasping forceps is necessary. However, some reports pointed out that routine prophylactic surgery is overtreatment in most cases and that it only increases the risk of surgery [14]. The management of cPPV remains controversial. In this study, 4.9% (44/901 unilateral inguinal hernias) of OR group developed CMIH. Expecting that 40% of 901 unilateral inguinal hernias in the OR group were hypothesized to have cPPVs (360 cases) by referring to this study of SILPEC, only around 12% (44/360) of cPPVs in the OR group would develop CMIH. Therefore, for 88% of patients with unilateral inguinal hernia, prophylactic surgery would be unnecessary. However, there is no way to predict the percentage of patients with cPPV who will develop CMIH at the present. Considering the low complication rate in experienced institutions, routine exploration and repair of cPPV could be a new standard method in this laparoscopic era. A randomized, prospective study with long-term follow-up period is required to confirm this in the future.

Generally, the cosmetic result is considered to be better in laparoscopic than in open surgery. However, in the traditional OR for inguinal hernia, inguinal crease incision is considered to be scarcely visible because it is along the skin crease and usually is concealed under the cloths. On the other hand, umbilical incision for laparoscopy can cause umbilical deformities like protrusion, especially in reduced port surgery [15]. Moreover, in this study, the incidence of SSI was significantly higher in SILPEC than in OR group. Only a few articles statistically analyzed the cosmetic result of these procedures [13]. To assess patients’ satisfaction with the cosmetic result, we sent the questionnaire by mail. In patients who underwent SILPEC without umbilicoplasty, only 7% of parents reported that the umbilicus developed a protrusion deformity, whereas 19% of parents felt that the umbilicus was better cosmetically due to our meticulous umbilical closure. Patients felt that the operative scar was more visible in OR than in SILPEC group, but both groups had an equally high level of satisfaction with operative scar. From the perspective of cosmetic result, both procedures produced satisfactory results. At the closure of the umbilicus after laparoscopic procedure, meticulous care should be taken not to leave umbilical deformity.

Laparoscopic approach for inguinal hernia is sometimes criticized to be performed unnecessarily in the abdominal cavity with a risk of intra-abdominal adhesions. During reoperation for the recurrent cases, none showed intra-abdominal adhesions, so laparoscopic approach has minimal risk of adhesions.

Our retrospective comparative study has a limitation in regards to the follow-up period to evaluate the long-term postoperative result, including recurrence and CMIH. As a matter of fact, long-term follow-up for all patients is difficult with a benign disease like inguinal hernia, as stated in previous studies. Therefore, we conducted a follow-up survey through sending questionnaires to the parents to confirm the clinical outcomes.

In conclusion, SILPEC and OR are comparable in terms of recurrence and other complications. SILPEC has the advantages of preventing CMIH and invisible scar. SILPEC proved to be safe and feasible in the midterm follow-up periods. SILPEC can be alternative to the traditional OR for pediatric inguinal hernia. The present data are limited by the short follow-up duration. A longer follow-up period would be expected to evaluate long-term outcomes as well as the incidence of infertility.
